# Supplementary material for: Activation and self-inactivation mechanisms of the cyclic oligoadenylate-dependent CRISPR ribonuclease Csm6
Source: Nat Commun. 2020 Mar 27;11:1596. doi: 10.1038/s41467-020-15334-5 (PMC7101355; doi:10.1038/s41467-020-15334-5)
Supplement: Supplementary file 2 — Peer Review File [file 41467_2020_15334_MOESM2_ESM.pdf]

Reviewers' comments:

Reviewer #1 (Remarks to the Author):

This manuscript answers two important questions about type III CRISPR systems, Namely, how does cOA6 activate Csm6 nuclease and how is this activation turned off. The first question is answered with a crystal structure of Csm6 bound to a cOA6 mimic. This structure provides insights into the conformational changes that drive Csm6 activation by cOA6 and into how coA6 is degraded, thus deactivating Csm6. Although structures of Csm6 bound to cOA4 have recently been reported, and cited appropriately here, this structural work is still important and provides a more complete picture of Csm6 regulation across species. Also, and importantly, the structural work is validated by both biochemical and genetic experiments. Overall this is a well written and illustrated manuscript and the conclusions are justified by the data provided. Given the interest in CRISPR biology this work should be of interest to a broad range of researchers

Reviewer #2 (Remarks to the Author):

In this manuscript, Garcia-Doval et al. describe the structural basis for activation and inactivation of the Csm6 ribonuclease from the <i>Enterococcus italicus</i> type III-A CRISPR-Cas system. During bacterial infection, Cas effector complexes from type III CRISPR-Cas systems generate cyclic oligoadenylate (cA) molecules upon binding to target RNA transcripts from the invading genome. These cA molecules act as activating signaling molecules by binding to the CARF domain of ancillary Csm6 or Csx1-family proteins that are encoded within the CRISPR-Cas locus. Upon cA binding, Csm6/Csx1 is activated as a non-specific RNase. Csm6 has also be shown to have ring nuclease activity, and can act to turn off the activation signal through the degradation of the cA molecule.

Here, the authors provide structural and mechanistic evidence on the activation of Csm6 by cA6, and the ring nuclease activity of Csm6 that cleaves cA6 and deactivates the protein. The authors solved a high-resolution structure of the Csm6-cA6 complex that provides important details on the conformation of the ligand in the CARF-domain active site and the conformational changes in the HEPN domain that result in RNase activation. The structural model indicates that the cA6 molecule is stabilized in a conformation that primes it for in-line attack of the scissile phosphate by the adjacent 2’-OH in the ribose backbone. Interestingly, the authors demonstrate that mutation of a threonine that stabilizes the transition state results in prolonged activation of Csm6, presumably by slowing or blocking degradation of the cA6.

Overall, this is an interesting study that answers key mechanistic questions about Csm6 activity in type III-A CRISPR-Cas immunity. The structure and biochemical experiments in the manuscript are very high quality, and I only have a few minor concerns. This work follows two other structural studies of Csm6 or Csx1 bound to cA4. However, the model for ring nuclease activity is better defined in the current manuscript. Given that this is the major new insight in the manuscript, the mechanism for the ring nuclease could potentially be supported with further evidence. Although I would not require additional experiments for this manuscript to be published, the authors could consider the suggestions below to further strengthen the models they present in the manuscript.

Experimental suggestions:
1. While the prolonged activation of the T11A mutant by cA6 is demonstrated through an assay measuring RNase activity, there is no direct evidence provided for how severely this mutant affects ring nuclease activity. The assay shown in Supplemental Fig. 1 could be used to demonstrate whether T11A completely abrogates cA6 degradation or only slows the activity.

2. The authors propose that the inactivation of Csm6 through its ring nuclease activity may prevent toxic self-cleavage activity. Have the authors tested the T11A mutant in their in vivo

system to determine whether prolonged Csm6 activity further slows bacterial growth?

Minor comments:
1. The authors do not provide a comparison of the activated HEPN domain with an unactivated structure of Csm6. Is there a structural difference between the two? If so, it would be helpful to show the structural changes that occur in the HEPN domain upon cA6 binding. If not, the authors should discuss this point. Considering the proposed model that apo Csm6 is in conformational equilibrium between active and inactive states, have previous crystal structures of apo Csm6 mainly been captured in the activated state? Consistently, the structures of apo and cA4-bound ToCsm6 in reference 21 did not have significant structural differences.

2. The authors do not discuss the activity of T11A observed in Figure 2d. It is interesting that the mutant appears to have stronger RNase activation than WT Csm6. Is this a significant difference? Is the activation stronger for T11A due to the lack of ring nuclease activity?

3. The references to Supplementary Fig. 1 in the first paragraph of the Results section do not match with the figure (for example, the reference to Supplementary Fig. 1b should likely be Supplementary Fig. 1d). It would be helpful to label the peaks in the mass spectra, as the m/z values are very small and difficult to discern. The authors could also provide the expected m/z for the cA6 (presumably 2x that of the tri-AMP species?).

4. It may be useful to provide a brief description of the fluorogenic RNase assays in the figure legend of Supplemental Fig. 2 (or to provide a cartoon of the assay) for the sake of readers who may be less familiar with the use of RNaseAlert to assess Csm6 activity. For example, the authors could explain in the figure legend that an increase in fluorescence indicates the cleavage of the reporter RNA, generating a fluorescent signal.

5. In the bottom panels of Supplementary Figure 5, it is a bit difficult to distinguish the light purple and light gray colors used for one set of protomers. Also, for the other protomer, it is unclear which protein is which. An alternate color scheme could help, along with providing the colors for each protein in the figure legend.

6. Starting at bottom of p. 5 "A single molecule of cFA6 is bound within a conserved pocket spanning the dimer interface of the CARF domains (Fig. 1), as predicted by earlier structural studies of Thermus thermophilus Csm6, and recent structures of Thermococcus onnurineus (T oCsm6) and Sulfolobus islandicus (SisCsm6) proteins bound to c-tetra-AMP²¹⁻²³." To my knowledge, there is no Csm6 in <i>S. islandicus</i> which contains type III-B systems. I believe the structure the authors are referring to from <i>S. islandicus</i> is a Csx1 structure bound to cA4 reported in reference #25. Please update.

Reviewer #3 (Remarks to the Author):

The CRISPR-Cas systems provide adaptive immunity against invading nucleic acids. In the type III CRISPR-Cas systems, cyclic oligoadenylate second messengers activate the Csm6 family nucleases, which contain the CARF and HEPN domains. In this study, Garcia-Doval et al. showed that Enteroccocus italicus Csm6 (EiCsm6) degrades cyclic hexa-AMP (cA6) using its CARF domain, and determined the crystal structure of EiCsm6 bound to a cA6 analog (cFA6). Their structural and functional data provide mechanistic insights into the cA6 recognition by the Csm6 CARF domain and the c6A-mediated regulation of the RNA degradation by the Csm6 HEPN domain. Overall, I find this manuscript suitable for publication in Nature Communications, if the authors address the following points.

1. It would be informative to include a structural comparison with the T. onnurineus Csm6-cA4

complex (Ref. 21) and the S. islandicus Csx1-cA4 complex (Ref. 25), in terms of their overall structures and the cyclic oligoadenylate recognition.

2. P6. "…, and recent structures of Thermococcus onnurineus (ToCsm6) and Sulfolobus islandicus (SisCsm6) proteins bound to c-tetra-AMP21-23." SisCsm6 should be SisCsx1 (or SiCsx1), and Ref. 25, rather than Ref. 23, should be cited.

3. P7. "To verify the functional contribution of Thr11 towards catalyzing cA6 degradation, we compared the cA6-stimulated RNase activities of WT and T11A EiCsm6 proteins." It seems better to directly examine the cA6 degradation by the EiCsm6 T11A mutant, as in Supplementary Figure 1.

4. Figure 1b and 1c. It would be informative to number each FA moiety in the cFA6 molecule. In addition to Figure 1c, it would be informative to provide an omit electron density map for the cFA6 molecule (2mFo – Fc or mFo – Fc) as an enlarged, stereo image in a supplementary figure.

5. Figure 2d and 2e. It would be better to examine similar mutants in vitro and in vivo as much as possible.

6. Supplementary Figure 2. It would be better to examine the effect of the T11A, HEPN/T11A and T114A mutations, in addition to the HEPN/T114A mutation. The RNase activities presented in the figure are those of WT Csm6 in the presence of products, after pre-incubating cA6 with different Csm6 proteins. Thus, "Csm6" should be indicated as "Pre-incubated Csm6" to avoid confusion. The cA6 product pre-incubated with the HEPN mutant stimulated the RNase activity less efficiently, as compared to the product pre-incubated with either no Csm6 or the HEPN/T114A mutant. Does this mean that the HEPN domain is partially involved in the cA6 degradation?

7. Supplementary Figure 3a and 3b. Unlike the sample with cFA6, the samples with cA6 exhibited high fluorescence signals even at the 0-s time point. Why did the authors use 1 nM Csm6 and 100 nM cA6, rather than 0.5 nM Csm6 and 10 nM cA6, as in Figure 2d (where increases in the fluorescence signals can be nicely monitored)? Does the result indicate that cA6 stimulates the Csm6 RNase activity more efficiently, as compared to cFA6?

8. There are some typos that should be fixed. T11 -> Thr11 (P9); 100'000 -> 100,000 (P11).

Reviewers' comments:

Reviewer #1 (Remarks to the Author):

This manuscript answers two important questions about type III CRISPR systems, Namely, how does cOA6 activate Csm6 nuclease and how is this activation turned off. The first question is answered with a crystal structure of Csm6 bound to a cOA6 mimic. This structure provides insights into the conformational changes that drive Csm6 activation by cOA6 and into how coA6 is degraded, thus deactivating Csm6. Although structures of Csm6 bound to cOA4 have recently been reported, and cited appropriately here, this structural work is still important and provides a more complete picture of Csm6 regulation across species. Also, and importantly, the structural work is validated by both biochemical and genetic experiments. Overall this is a well written and illustrated manuscript and the conclusions are justified by the data provided. Given the interest in CRISPR biology this work should be of interest to a broad range of researchers.

We thank the Reviewer for the positive assessment of our work.

Reviewer #2 (Remarks to the Author):

In this manuscript, Garcia-Doval et al. describe the structural basis for activation and inactivation of the Csm6 ribonuclease from the Enterococcus italicus type III-A CRISPR-Cas system. During bacterial infection, Cas effector complexes from type III CRISPR-Cas systems generate cyclic oligoadenylate (cA) molecules upon binding to target RNA transcripts from the invading genome. These cA molecules act as activating signaling molecules by binding to the CARF domain of ancillary Csm6 or Csx1-family proteins that are encoded within the CRISPR-Cas locus. Upon cA binding, Csm6/Csx1 is activated as a non-specific RNase. Csm6 has also be shown to have ring nuclease activity, and can act to turn off the activation signal through the degradation of the cA molecule.

Here, the authors provide structural and mechanistic evidence on the activation of Csm6 by cA6, and the ring nuclease activity of Csm6 that cleaves cA6 and deactivates the protein. The authors solved a high-resolution structure of the Csm6-cA6 complex that provides important details on the conformation of the ligand in the CARF-domain active site and the conformational changes in the HEPN domain that result in RNase activation. The structural model indicates that the cA6 molecule is stabilized in a conformation that primes it for in-line attack of the scissile phosphate by the adjacent 2’-OH in the ribose backbone. Interestingly, the authors demonstrate that mutation of a threonine that stabilizes the transition state results in prolonged activation of Csm6, presumably by slowing or blocking degradation of the cA6.

Overall, this is an interesting study that answers key mechanistic questions about Csm6 activity in type III-A CRISPR-Cas immunity. The structure and biochemical experiments in the manuscript are very high quality, and I only have a few minor concerns. This work follows two other structural studies of Csm6 or Csx1 bound to cA4. However, the model for ring nuclease activity is better defined in the current manuscript. Given that this is the major new insight in the manuscript, the mechanism for the ring nuclease could potentially be supported with further evidence. Although I would not require additional experiments for this manuscript to be published, the authors could consider the suggestions below to further strengthen the models they present in the manuscript.

We thank the Reviewer for the positive evaluation of our work and for the constructive comments, which have helped us to strengthen the manuscript. We hope that the additional experiments and revisions to the manuscript, as detailed below, have made it acceptable for publication.

Experimental suggestions:
1. While the prolonged activation of the T11A mutant by cA6 is demonstrated through an assay measuring RNase activity, there is no direct evidence provided for how severely this mutant affects ring nuclease activity. The assay shown in Supplemental Fig. 1 could be used

to demonstrate whether T11A completely abrogates cA6 degradation or only slows the activity.

We agree with the Reviewer and have now performed in vitro experiments to check the ring nuclease activity of the T11A mutant. The experiments are shown in Figures S6a and S6b. Our results indicate that the T11A mutation substantially decreases the ring nuclease activity, but it is not sufficient to abolish it completely. This is consistent with the proposed catalytic mechanism of cA6 degradation, in which Thr11 is presumed to play a role in stabilizing the transition state but is not involved in acid-base catalysis of the reaction.

2. The authors propose that the inactivation of Csm6 through its ring nuclease activity may prevent toxic self-cleavage activity. Have the authors tested the T11A mutant in their in vivo system to determine whether prolonged Csm6 activity further slows bacterial growth?

We agree with the Reviewer and have now tested the effect of the T11A mutation in vivo under two experimental conditions; the data is shown in Figures 2E and 3C.

In the presence of a Cas10-Csm effector complex containing a dHD mutation (wild-type Csm3), the T11A mutation reduces the Csm6-dependent toxicity (growth inhibition) phenotype upon induction of target transcription with 12.5 ng/ml aTc (Fig. 2e). We believe that this is because the physiological levels of cA6 generated by the Cas10-Csm complex during the response are insufficient to fully activate the T11A Csm6 mutant because its affinity for cA6 is reduced.

In type III systems, cA6 signaling is ultimately terminated by the RNase activity of the Csm3 subunits of the Cas10-Csm effector complex, which catalyze cleavage of the RNA target and thereby inactivate the Cas10 Palm domain to stop cA6 production. When target transcription is induced with a low concentration of aTc (5 ng/ml), and the RNase activity of the Cas10-Csm complex is simultaneously abolished by a point mutation in the Csm3 subunit (dCsm3), expression of WT Csm6 does not result in a growth arrest phenotype. In contrast, expression of the T11A Csm6 mutant results in greatly enhanced cell toxicity and a growth inhibition phenotype under these conditions, suggesting that Csm6 T11A is hyperactive when cA6 production is persistent. This result is in agreement with the in vitro data shown in Fig. 3b and corroborates our structural data implicating Thr11 in the ring nuclease activity of Csm6. Furthermore, this experiment provides strong evidence that the ring nuclease of Csm6 limits self-toxicity during CRISPR interference.

Minor comments:

1. The authors do not provide a comparison of the activated HEPN domain with an unactivated structure of Csm6. Is there a structural difference between the two? If so, it would be helpful to show the structural changes that occur in the HEPN domain upon cA6 binding. If not, the authors should discuss this point. Considering the proposed model that apo Csm6 is in conformational equilibrium between active and inactive states, have previous crystal structures of apo Csm6 mainly been captured in the activated state? Consistently, the structures of apo and cA4-bound ToCsm6 in reference 21 did not have significant structural differences.

Of the Csm6/Csx1 structures determined to date, only ToCsm6 and SisCsx1 have been crystallized both in the presence and absence of cA4. In both cases, the respective apo- and cA4-bound proteins were crystallized in the same crystal system (same space group and near-identical unit cell constants) and the rmsd deviations between the apo- and cA4-bound structures are quite small (~0.6 Å for ToCsm6 and ~0.8-0.9 Å for SisCsx1). Similarly, in our case, the apo-EiCsm6 structure (for we were able to build a model but could not refine it to an acceptable Rfree factor) is nearly identical with that of the cA6-bound complex.

Although Molina et al. maintain that the apo and cA4 SisCsx1 structures provide an explanation for cA4-induced activation of the HEPN domains in terms of the observed conformational differences, we do not believe that the structural differences are significant enough to warrant this conclusion as they are likely within the coordinate error at the resolution at which the structures have been determined or might be simply due to crystal packing.

Collectively, these structures indicate that the HEPN domain conformations are essential identical in both the apo and cOA-bound states, thus suggesting that the apo-Csm6/Csx1 structures have been captured in the activated state. This would imply a mechanistic model in which apo-Csm6/Csx1 proteins dynamically sample a conformational equilibrium, and that cA4 or cA6 binding shifts the equilibrium towards the activated state by preferentially stabilizing it, i.e. a model based on conformational selection.

Further experimental and computational studies will be required to test this hypothesis. We are currently attempting to investigate the conformational dynamics of EiCsm6 using both bulk and single-molecule FRET studies, but these experiments are currently beyond the scope and timeframe of this manuscript.

2. The authors do not discuss the activity of T11A observed in Figure 2d. It is interesting that the mutant appears to have stronger RNase activation than WT Csm6. Is this a significant difference? Is the activation stronger for T11A due to the lack of ring nuclease activity?

Although the T11A does seems to have a slightly higher activity in the cA6 activation experiment in Fig. 2d, we do not think that the activity is significantly different to justify this conclusion, given that the WT and T11A proteins used are different samples whose specific activities may be different due to slight differences in purity or different fractions of the protein being inactive.

Our in vivo assays (added during the revision, shown in Fig. 2e) show that T11A displays lower activity in vivo, which we attribute to partially impaired cA6 binding and resulting reduced allosteric activation. Under the experimental conditions used for the in vitro assay (Fig. 2d), the T11A mutant is active as the cA6 concentration is likely to be higher than its dissociation constant for T11A Csm6.

3. The references to Supplementary Fig. 1 in the first paragraph of the Results section do not match with the figure (for example, the reference to Supplementary Fig. 1b should likely be Supplementary Fig. 1d). It would be helpful to label the peaks in the mass spectra, as the m/z values are very small and difficult to discern. The authors could also provide the expected m/z for the cA6 (presumably 2x that of the tri-AMP species?).

We thank the Reviewer for spotting the mistake. We have corrected the figure references throughout the text.

We have added labels in Fig. S1 next to the peaks indicating the measured m/z values. We also included an extra panel (g) summarizing the calculated theoretical m/z for cA6, cA3 and cA2. Although the mechanism predicts A2>P and A3>P as reaction products, we cannot differentiate the linear and cyclic version based on their m/z, as we described in our previous work for cA6. For clarity, we have also added the m/z in Fig. S3 and the expected m/z in the corresponding figure legend. We also included this information in the new Fig. S6.

4. It may be useful to provide a brief description of the fluorogenic RNase assays in the figure legend of Supplemental Fig. 2 (or to provide a cartoon of the assay) for the sake of readers who may be less familiar with the use of RNaseAlert to assess Csm6 activity. For example, the authors could explain in the figure legend that an increase in fluorescence indicates the cleavage of the reporter RNA, generating a fluorescent signal.

We have added a brief description of the fluorogenic RNase assay in Fig. S2a.

5. In the bottom panels of Supplementary Figure 5, it is a bit difficult to distinguish the light purple and light gray colors used for one set of protomers. Also, for the other protomer, it is unclear which protein is which. An alternate color scheme could help, along with providing the colors for each protein in the figure legend.

We have adjusted the colors used in the structural superpositions (now Fig. S7a) to increase the contrast between the gray and blue. We have also added the color information in the figure legend.

6. Starting at bottom of p. 5 “A single molecule of cFA6 is bound within a conserved pocket spanning the dimer interface of the CARF domains (Fig. 1), as predicted by earlier structural studies of Thermus thermophilus Csm6, and recent structures of Thermococcus onnurineus (T oCsm6) and Sulfolobus islandicus (SisCsm6) proteins bound to c-tetra-AMP[21-23]." To my knowledge, there is no Csm6 in S. islandicus which contains type III-B systems. I believe the structure the authors are referring to from S. islandicus is a Csx1 structure bound to cA4 reported in reference #25. Please update.

We thank the Reviewer for spotting the error. We have corrected Csm6 to Csx1.

Reviewer #3 (Remarks to the Author):

The CRISPR-Cas systems provide adaptive immunity against invading nucleic acids. In the type III CRISPR-Cas systems, cyclic oligoadenylate second messengers activate the Csm6 family nucleases, which contain the CARF and HEPN domains. In this study, Garcia-Doval et al. showed that Enteroccocus italicus Csm6 (EiCsm6) degrades cyclic hexa-AMP (cA6) using its CARF domain, and determined the crystal structure of EiCsm6 bound to a cA6 analog (cFA6). Their structural and functional data provide mechanistic insights into the cA6 recognition by the Csm6 CARF domain and the c6A-mediated regulation of the RNA degradation by the Csm6 HEPN domain. Overall, I find this manuscript suitable for publication in Nature Communications, if the authors address the following points.

We thank the Reviewer for the evaluation of our work and for the helpful comments. We have made our best efforts to address these points and hope that the revisions have made the manuscript acceptable for publication.

1. It would be informative to include a structural comparison with the T. onnurineus Csm6-cA4 complex (Ref. 21) and the S. islandicus Csx1-cA4 complex (Ref. 25), in terms of their overall structures and the cyclic oligoadenylate recognition.

We have included structural superpositions of the CARF domains of EiCsm6 with those of ToCsm6 and SisCsx1 as Fig. S4c and make comparisons in the text on p. 6.

2. P6. “…, and recent structures of Thermococcus onnurineus (ToCsm6) and Sulfolobus islandicus (SisCsm6) proteins bound to c-tetra-AMP21-23.” SisCsm6 should be SisCsx1 (or SiCsx1), and Ref. 25, rather than Ref. 23, should be cited.

We thank the Reviewer for spotting the errors. SisCsm6 has been changed to SisCsx1 and the reference has been corrected.

3. P7. “To verify the functional contribution of Thr11 towards catalyzing cA6 degradation, we compared the cA6-stimulated RNase activities of WT and T11A EiCsm6 proteins.” It seems better to directly examine the cA6 degradation by the EiCsm6 T11A mutant, as in Supplementary Figure 1.

We agree with the Reviewer about the importance of comparing the in vitro cA6 degradation activity of T11A Csm6 mutant with WT Csm6 (or the dHEPN mutant). We have investigated the activities using both the cA6 preincubation assay (Fig. S6a) and by LC-MS (Fig. S6b).

4. Figure 1b and 1c. It would be informative to number each FA moiety in the cFA6 molecule. In addition to Figure 1c, it would be informative to provide an omit electron density map for the cFA6 molecule (2mFo – Fc or mFo – Fc) as an enlarged, stereo image in a supplementary figure.

We have generated a stereo figure (Fig. S4a) showing an enlarged view of the 2mFo-Fc electron density corresponding to the cFA6 ligand.

5. Figure 2d and 2e. It would be better to examine similar mutants in vitro and in vivo as much as possible.

We have consolidated the mutagenesis experiments and now show in vitro (Fig. 2d) and in vivo (Fig. 2e) mutant data for the same set of CARF domain residues.

6. Supplementary Figure 2. It would be better to examine the effect of the T11A, HEPN/T11A and T114A mutations, in addition to the HEPN/T114A mutation. The RNase activities presented in the figure are those of WT Csm6 in the presence of products, after pre-incubating cA6 with different Csm6 proteins. Thus, “Csm6” should be indicated as “Pre-incubated Csm6” to avoid confusion. The cA6 product pre-incubated with the HEPN mutant stimulated the RNase activity less efficiently, as compared to the product pre-incubated with either no Csm6 or the HEPN/T114A mutant. Does this mean that the HEPN domain is partially involved in the cA6 degradation?

We agree with the Reviewer that these experiments would provide extra information and we now included them (as outlined in our reply to Reviewer’s point #3) as Fig. S6a. Fig. S2b was adjusted to include more data points. Experiments shown in Fig. S2b and S6a were run in parallel using the same controls (WT, dHEPN, cA6), but the T11A data are shown separately (Fig. S6a) for clarity.

The results of the experiments indicate that the HEPN domain contributes to cA6 degradation in vitro, which is not surprising given its non-specific ribonuclease activity. However, this is most likely a trans-acting cA6 nuclease activity, as opposed to the cis-acting ring nuclease activity of the CARF domain.

7. Supplementary Figure 3a and 3b. Unlike the sample with cFA6, the samples with cA6 exhibited high fluorescence signals even at the 0-s time point. Why did the authors use 1 nM Csm6 and 100 nM cA6, rather than 0.5 nM Csm6 and 10 nM cA6, as in Figure 2d (where increases in the fluorescence signals can be nicely monitored)? Does the result indicate that cA6 stimulates the Csm6 RNase activity more efficiently, as compared to cFA6?

We used higher concentrations in the experiments because cFA6 is a weaker activator compared to cA6, presumably because it binds less strongly to the CARF domain. This is consistent with the structural data showing that the 2’-F groups in cFA6 (and therefore presumably the 2’-OH groups in cA6) are directly contacted by the CARF domain. In order to clarify this point, we have measured the EC_{50} for cFA6 (~170 nM; now shown in Fig. S3d), which confirms that it is a weaker activator than cA6 (EC_{50} ~3.5 nM).

8. There are some typos that should be fixed. T11 -> Thr11 (P9); 100’000 -> 100,000 (P11).

We thank the Reviewer for spotting the typos. We have corrected them.

REVIEWERS' COMMENTS:

Reviewer #2 (Remarks to the Author):

Garcia-Doval et al. report the structural mechanism for activation and inactivation of the RNase Csm6 by the cA6 signaling molecule produced in type III-A CRISPR-Cas systems. The high-quality structural, biochemical and in vivo data in this manuscript provide the most extensive mechanistic model for the activity of Csm6 in CRISPR-based immunity. In particular, the authors provide a compelling model for the basis of ring nuclease by the Csm6 CARF domain, which leads to self-inactivation following a short burst of non-specific RNase activity. In the revised manuscript, the authors have included several new experiments based on suggestions by the reviewers that strengthen their model for this inactivation. Overall, this is an excellent study and I strongly recommend publication as is, with a few minor adjustments.

Minor concerns:
1. For consistency, please change the color of T11A data in Figure 2e to red.
2. In the figure legend for Supplementary Figure 6b, I believe "T11 EiCsm6" should be "T11A EiCsm6".

Reviewer #3 (Remarks to the Author):

The authors have addressed my previous concerns, and the manuscript have been improved. I have a minor comment about Supplementary Figure 4a, where the stereo figure should have a width of 11 cm (equivalent points separated by 5.5 cm), as stated in the checklist, and should contain labels in the both panels.

Manuscript: NCOMMS-19-32153B

Carcia-Doval et al.

RESPONSE to REVIEWERS' COMMENTS:

Reviewer #2 (Remarks to the Author):

Garcia-Doval et al. report the structural mechanism for activation and inactivation of the RNase Csm6 by the cA6 signaling molecule produced in type III-A CRISPR-Cas systems. The high-quality structural, biochemical and in vivo data in this manuscript provide the most extensive mechanistic model for the activity of Csm6 in CRISPR-based immunity. In particular, the authors provide a compelling model for the basis of ring nuclease by the Csm6 CARF domain, which leads to self-inactivation following a short burst of non-specific RNase activity. In the revised manuscript, the authors have included several new experiments based on suggestions by the reviewers that strengthen their model for this inactivation. Overall, this is an excellent study and I strongly recommend publication as is, with a few minor adjustments.

We thank the Reviewer for the positive comments.

Minor concerns:
1. For consistency, please change the color of T11A data in Figure 2e to red.

The color has been changed.

2. In the figure legend for Supplementary Figure 6b, I believe “T11 EiCsm6” should be “T11A EiCsm6”.

The error has been corrected.

Reviewer #3 (Remarks to the Author):

The authors have addressed my previous concerns, and the manuscript have been improved. I have a minor comment about Supplementary Figure 4a, where the stereo figure should have a width of 11 cm (equivalent points separated by 5.5 cm), as stated in the checklist, and should contain labels in the both panels.

We thank the Reviewer for the comment. We have corrected the final size of the stereo figure in the Supplementary Information File.
